# Patients with the most advanced rheumatoid arthritis remain with Th1 systemic defects after TNF inhibitors treatment despite clinical improvement

**DOI:** 10.1007/s00296-013-2895-9

**Published:** 2013-11-13

**Authors:** Agata Kosmaczewska, Jerzy Swierkot, Lidia Ciszak, Aleksandra Szteblich, Agnieszka Chrobak, Lidia Karabon, Anna Partyka, Jacek Szechinski, Piotr Wiland, Irena Frydecka

**Affiliations:** 1 Department of Immunopathology, Institute of Immunology and Experimental Therapy, Polish Academy of Sciences, R. Weigla 12, 53-114 Wrocław, Poland; 2 Department of Rheumatology and Internal Medicine, Wroclaw Medical University, Wrocław, Poland

**Keywords:** Rheumatoid arthritis, Th1/Th17/Treg imbalance, Cytokines, MTX, TNF inhibitors

## Abstract

Systemic immune defects might reflect severely dysregulated control of chronic inflammation related to disease progression. Th17/Treg cell imbalance has been demonstrated to be involved in rheumatoid arthritis (RA) pathogenesis. Despite controversial results, a growing anti-inflammatory role in this process has been recently attributed to Th1 responses. The aim of the study was to estimate the extent of Th1/Th17/Treg imbalance in peripheral blood (PB) of patients with short- and long-term RA in relation to cytokine milieu and its reversal after therapy with methotrexate and/or TNF inhibitors, respectively. Patients with different duration of RA (median 6 vs. 120 months) in the active phase of RA were enrolled in this study. We performed flow cytometric analysis of PB Th1, Th17, and Treg populations together with estimation of serum cytokine concentrations using cytometric bead array. Disease activity was calculated on the basis of clinical and biochemical indices of inflammation (DAS28, ESR, CRP). All parameters were measured and correlated with each other before and after 6 months therapy. Elevated levels of circulating Th17 cells and IL-6 were found in all active patients, of which Th17 cells were down-regulated by the treatment. Significantly reduced Th1 and functional CTLA-4+ Treg cell frequencies as well as Th1 cytokines observed only in progressive RA seemed to be irreversible. Although therapy induced clinical improvement in almost all patients, those with advanced RA remained with signs of inflammation. Our report demonstrates that both the extent of systemic immune abnormalities and their restoration are dependent on duration of the active RA.

## Introduction

Rheumatoid arthritis (RA) is one of the most common systemic autoimmune diseases. It is characterized by chronic inflammation in the synovium, resulting in progressive destruction of joints and cartilage. The persistent nature of arthritis strongly suggests immune dysfunctions associated with predominance of the pro-inflammatory responses. It seems that both local and systemic immune abnormalities may be involved in the disease development and evolution. Helper T cells (Th cells) play a major role in initiation and maintenance of the chronic inflammation in RA [1]. According to the cytokine microenvironment, CD4 T cells differentiate toward various pro- and anti-inflammatory subpopulations, including Th1, Th2, Th17, and T regulatory (Treg) cells, playing important roles in the pathogenesis of RA [2].

It has been shown that active RA results from an imbalance in the distribution of pro-inflammatory Th17 and anti-inflammatory Treg cells, which emphasizes the crucial roles of these cells in controlling RA [3]. Th1/Th17/Treg subsets can act as important participants of the complex network of interactions that manage the development and progression of RA, and, what is more important, they can exhibit this action at different stages of the disease with different intensities. Recent data have suggested interferon-gamma (IFN-γ) and/or interleukin-2 (IL-2) to be protective rather than pro-inflammatory cytokines that could be involved in the down-regulation of RA-related chronic inflammation [4, 5]. In light of the controversial results, the exact impact of Th1 cell responses on Th17/Treg cell systemic distribution in RA remains uncertain [6].

Therefore, we performed a comparative analysis of Th1/Th17/Treg cell distribution in relation to cytokine milieu in peripheral blood (PB) of patients divided into two groups with regard to RA duration (early and advanced stages of RA). Then, we evaluated modifications of studied parameters after 6 months of therapy with methotrexate (MTX) and/or tumor necrosis factor inhibitors (iTNF). We also correlated the results with clinical and laboratory markers of inflammation (DAS28, CRP, ESR) at each time point tested. All results were compared to those obtained in healthy controls.

## Patients and methods

### Ethics statement

The study was approved by the Ethics Committee at Wroclaw Medical University, Poland. Written informed consent was obtained from each patient and healthy donor after a full explanation of the procedure, according to the Declaration of Helsinki.

### Patients

The main characteristics of RA patients are summarized in Table 1. Forty-four patients with RA fulfilling the 1987 diagnostic criteria of the American Rheumatism Association (ARA) [7] and 13 healthy volunteers were included in the study. Eight patients were excluded from the analysis due to irregular reporting to follow up visits and incomplete data regarding activity of the disease and adverse effects. In the final study, 19 patients received oral MTX at a dose of 15 mg per week (MTX group); median (interquartile range) RA duration was 6 months (3–16 months). The other 17 were treated with recommended doses of TNF-α inhibitors (infliximab (*n* = 11), adalimumab (*n* = 2), or etanercept (*n* = 4) (iTNF group); median RA duration was 120 months (60–160 months). One of the inclusion criteria was active form of the disease established on the basis of clinical and biochemical indices of inflammation, such as the erythrocyte sedimentation rate (ESR), C-reactive protein (CRP), and calculation of the disease activity score (DAS28); for MTX treatment DAS28 > 3.2, and for iTNF administration DAS28 > 5.1. Based on the statistically significant differences in the median active disease duration and DAS28 score either before or after the treatment between the two groups, iTNF patients were assigned to the progressive form of the disease.

**Table 1:** Patients’ characteristics before and after therapy with MTX and/or iTNF

	MTX therapy group			iTNF therapy group			MTX versus iTNF therapy (*P* value)	
	Before	After	*P* value	Before	After	*P* value	Before	After
Number (*n*)	19			17				
MTX^a^ treatment +/−		19/19		13/17	13/17			
INF^b^ treatment +/−					11/17			
ADA^c^ treatment +/−					2/17			
ETA^d^ treatment +/−					4/17			
Sex (F/M)	12/7			14/3				
Age (years)	55.0 [41.5–67.0]			52.5 [46.0–55.0]			NS	
Duration (months)	6.0 [3.0–15.9]			120.0 [60.0–160.0]			0.0000001	
DAS 28	5.8 [4.9–6.2]	3.5 [3.1–4.0]	0.0000001	6.1 [5.7–6.9]	4.7 [4.0–5.8]	0.00007	0.02	0.0001
TJC	10.0 [8.0–14.0]	2.0 [1.0–4.0]	0.00006	14.0 [11.0–18.0]	7.0 [5.0–11.0]	0.00003	0.03	0.0002
SJC	6.0 [4.0–10.0]	1.0 [0.0–2.0]	0.00009	8.5 [6.0–13.0]	4.0 [3.0–6.0]	0.0003	NS	0.0002
VAS (mm) (%)	60.0 [47.0–70.0]	23.0 [10.0–30.0]	0.0000001	67.5 [63.0–80.0]	40.0 [25.0–60.0]	0.0007	0.02	0.001
ESR (mm/h)	28.0 [17.5–39.0]	18.0 [12.0–26.0]	0.006	26.0 [19.0–44.0]	21.0 [17.0–33.0]	NS	NS	NS
CRP (mg/L)	11.0 [6.5–19.0]	3.6 [2.1–6.8]	0.005	15.8 [10.0–31.0]	8.0 [4.1–42.0]	NS	NS	0.05
Leukocytes (G/L)	7.7 [6.0–9.3]	6.7 [4.9–7.9]	0.009	9.6 [6.8–11.5]	8.9 [6.1–10.6]	NS	0.05	NS
Platelets (x10^9^/L)	304.0 [220.0– 348.0]	249.0 [200.0–298.0]	0.02	294.0 [260.0–377.0]	285.5 [242.00–353.00]	NS	NS	NS
Hb (g/dL)	12.2 [11.8–13.5]	12.7 [11.8–14.0]	NS	12.7 [11.9–12.9]	12.9 [12.4–13.5]	NS	NS	NS
% CD4^+^ lymphocytes in total T cells	63.9 [52.6–78.2]	59.7 [50.4–63.3]	NS	71.9 [60.2–77.4]	67.4 [59.9–69.6]	NS	NS	NS

DAS28, disease activity score rated by the 28 joint count; TJC, tender joint count; SJC, swollen joint count; VAS, general health patient; ESR, erythrocyte sedimentation rate; CRP, C-reactive protein

^a^MTX: up to 25 mg/week orally

^b^INF: 3 mg/kg body weight; i.v. infusion at weeks: 0, 2, and 6, and every 8 weeks thereafter

^c^ADA: 40 mg every other week; s.c.

^d^ETA: 50 mg every week; s.c.

For biologics to be used, treatment failure with at least two traditional disease-modifying antirheumatic drugs (DMARDs) including MTX was required. Thus, all iTNF patients had received MTX in the past history; 13 out of 17 patients were still receiving MTX when iTNF therapy was started. Because of the disease activity, patients were allowed to continue treatment with DMARDs, including MTX, sulphasalazine, glucocorticoids, and nonsteroidal anti-inflammatory drugs, if the treatment regimens were not modified 4 weeks before the study. Based on the efficacy of the therapy, the clinical improvement of RA was established according to the response criteria suggested by the European League Against Rheumatism (EULAR), if reduction of DAS28 was >0.6 (moderate efficacy) or even >1.2 (good efficacy)[8]. Accordingly, at least partial improvement was achieved in 14 (82.35 %) and 19 (100 %) patients treated with iTNF and MTX, respectively. Blood samples were collected just before and after 6 months of the therapy. Healthy individuals were free of the following events: inflammatory syndrome (CRP < 0.5 mg/dL), steroid or immunosuppressive therapy, acute or chronic infectious diseases, other autoimmune diseases, and cancers. The controls were matched with patients for age and sex.

### Cell preparation, culture conditions, and flow cytometry

Peripheral blood mononuclear cells (PBMCs) were isolated by buoyant density-gradient centrifugation on Lymphoflot (Biotest, Germany) from 40 ml of freshly drawn peripheral venous blood (PB). Because incubation with phorbol 12-myristate 23-acetate (PMA) triggers internalization and degradation of the CD4 molecule, which would affect the identification of Th1 (CD4+IL-17-IFN-γ+) and Th17 (CD4+IFN-γ-IL-17+) cells [9], CD4+ T cells were negatively separated before cytokine staining. CD4+ T cells purified by magnetic cell sorting (Miltenyi Biotec) were then stimulated with 25 ng/ml PMA and 1 μg/ml of ionomycin (Ion) (Sigma-Aldrich) in the presence of 10 μg/ml of brefeldin A (BFA, protein transport inhibitor) for 4 h at 37 °C in a humidified atmosphere containing 5 % CO_2_. For analysis of Tregs (CD4+FoxP3+ T cells) as well as CTLA-4+Tregs, PBMCs were aliquoted into tubes directly after isolation for further staining performed as follows: the cells were stained with anti-CD3/FITC (clone UCHT1; BD Pharmingen), anti-CD4/PerCP (clone SK3; BD Pharmingen) or anti-CD4/FITC (clone SK3; BD Pharmingen), and CTLA-4/PE-Cy monoclonal antibodies (mAbs) (clone BNI3; BD Pharmingen). For intracellular staining, the cells were fixed and permeabilized with BD Permeabilizing Solution 2 (Becton–Dickinson) according to the manufacturer’s instructions with subsequent incubation with anti-IFN-γ/FITC (clone 25723.11; BD Pharmingen), anti-IL-17/PE (clone eBio64DEC17; eBioscience), anti-human FoxP3/PE mAbs (clone 259D/C7; BD Pharmingen), or fluorochrome-labeled isotypic control mAbs for 30 min at room temperature in the dark. Directly after immunostaining, the cells were washed and analyzed by flow cytometry using a FACScan cytometer equipped with Cell Quest software (BD Bioscience).

### RNA isolation and real-time PCR (RT-PCR)

Total RNA was extracted from CD4+ T cells using RNeasy columns (Qiagen, Hilden, Germany), subjected to DNase I treatment according to the manufacturer’s protocols and immediately 800 ng of RNA was reverse transcribed using the iScript cDNA Synthesis Kit (Biorad, Hercules, CA, USA). The mRNA levels of human IL-17A, FoxP3, INFγ, and β2 microglobulin (β2M) were analyzed by quantitative real-time PCR using primers and probes from Applied Biosystems (Hs00174383_m10, Hs01085832_m1, Hs00989291_m1, and Human β2M, respectively). All the samples were analyzed using the 7300 Real-Time Instrument (Applied Biosystems). To calculate relative expression, all results were analyzed according to the Δ*C*_*t*_ method using β2M as a reference gene.

### Cytokine assays

Cytokines (IL-6, IL-2, IFN-γ, TNF-α, and IL-17) were measured in patients’ and controls’ sera by a flow cytometric bead array using human cytokines kits of the BD™ CBA Human Soluble Protein Flex Set system (Becton–Dickinson) and analyzed on a FACSCalibur flow cytometer (Becton–Dickinson), as recently described [10].

### Statistical analysis

One-way ANOVA test was used to determine significant differences between groups. Spearman’s test was used for correlation analysis. The Wilcoxon signed rank test was used to compare paired patients before and after the treatment. Results were considered statistically significant when *P* ≤ 0.05. Data are presented as median (interquartile range). Statistica’99 Edition was used in the statistical calculations.

## Results

### Pre-treatment distribution of PB Th1, Th17, and Treg cell population

**Fig. 1:**
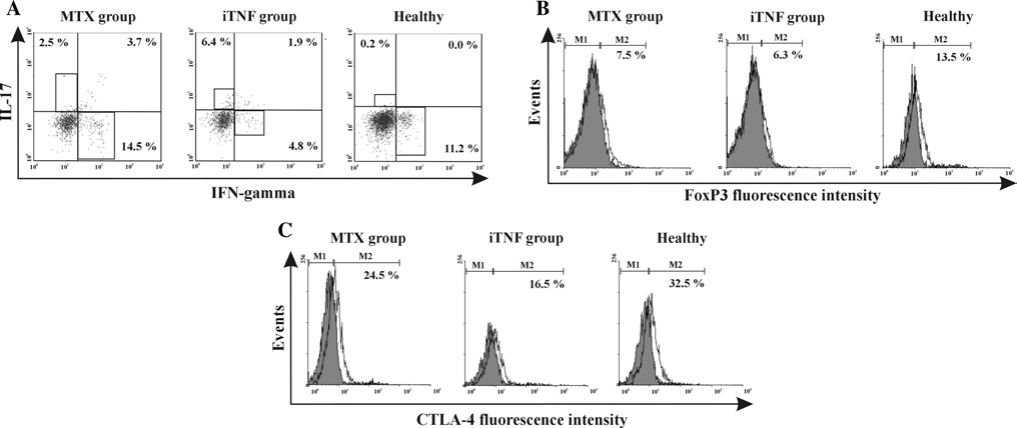
Representative examples of flow cytometric analyses of Th1, Th17, Treg, and functional CTLA-4+ Treg cell populations in active RA patients and controls. **a** Th1 (CD4+IL-17-IFN-γ+) and Th17 (CD4+IFN-γ-IL-17+) cells in studied groups presented as dot plots. **b** Expression of FoxP3 transcription factor in total CD4+ T cells in the studied groups presented as open histograms. **c** Expression of CTLA-4 suppressive molecule in total CD4+FoxP3+ T cells in the studied groups presented as open histograms. *Grey histograms* represent isotype controls

**Fig. 2:**
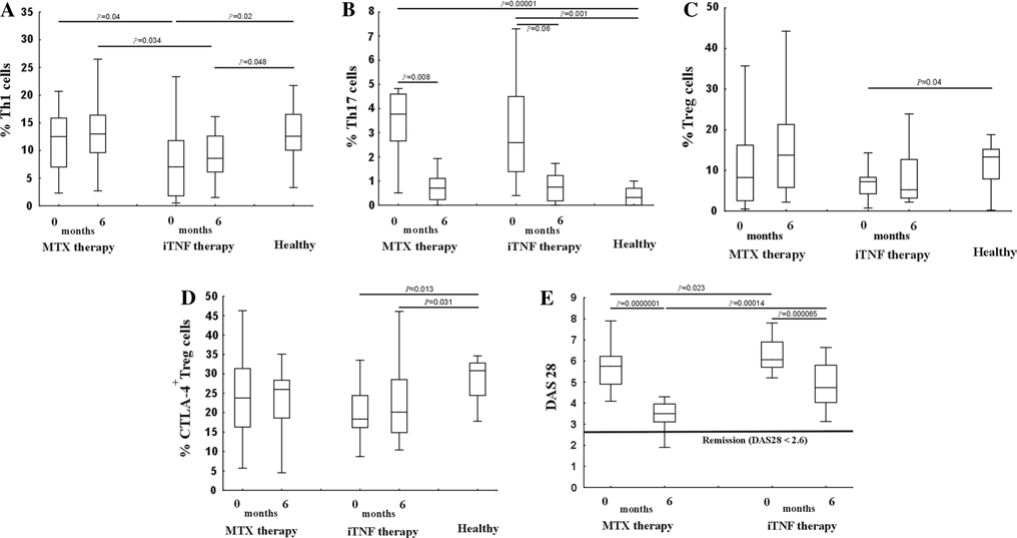
Frequencies of circulating Th1, Th17, Treg, and CTLA-4+ Treg cells and DAS28 in RA patients before and after therapy compared to healthy controls. **a** Th1 (CD4+IL-17-IFN-γ+) cells. **b** Th17 (CD4+IFN-γ-IL-17+) cells. **c** Treg (CD3+CD4+Foxp3+) cells. **d** Functional Treg (CD4+Foxp3+CTLA-4+) cells. **e** Modification of the DAS28. Whiskers are as follows: median (*horizontal bar*), IQ range (*box*), and extreme values (*error bars*)

In order to determine the relationship between the duration of active RA and distribution of anti- and pro-inflammatory Th cell subpopulations in PB, we assessed the proportion of Th1, Th17, and Treg cells in patients before MTX and/or iTNF administration. We did not find any significant differences in the relative mRNA expression of IFN-γ, IL-17, and FoxP3 between all studied groups (Table 2). Both groups of RA patients exhibited significantly and similarly increased Th17 populations compared to controls (Figs. 1a, 2b). Only iTNF patients possessed a reduced Th1 cell population compared to others. There were no differences in Th1 cell frequency in PB between MTX patients and controls (Figs. 1a, 2a). The proportions of circulating Treg cells as well as those co-expressing the suppressor CTLA-4 molecule (CTLA-4+ Tregs) were lower in the iTNF group than in controls, in contrast to the similar values in the MTX group and healthy subjects (Figs. 1b, c, 2c, d).

**Table 2:** IFN-γ, Foxp3, and IL-17 mRNA relative expression in PB CD4+ T cells of RA patients and normal controls; the median levels of mRNA [interquartile range]

	IFN mRNA	Foxp3 mRNA	IL-17 mRNA		
**MTX group**					
Before therapy	1.81 [1.53–3.21]	0.004 [0.002–0.006]	0.027 [0.014–0.048]		
After therapy	1.59 [1.34–2.67]	0.003 [0.001–0.004]	0.016* [0.011–0.036]		
**iTNF group**					
Before therapy	1.5 [0.55–2.17]	0.003 [0.001–0.004]	0.021** [0.07–0.034]		
After therapy	1.53 [0.71–2.5]	0.003 [0.001–0.005]	0.036 [0.018–0.052]		
Control group	1.4 [0.76–1.73]	0.001 [0.001–0.002]	0.017*** [0.011–0.018]		

* *P* = 0.048, ** *P* = 0.007, *** *P* = 0.01  compared to the iTNF group after therapy

### Pre-treatment serum cytokine profile

**Fig. 3:**
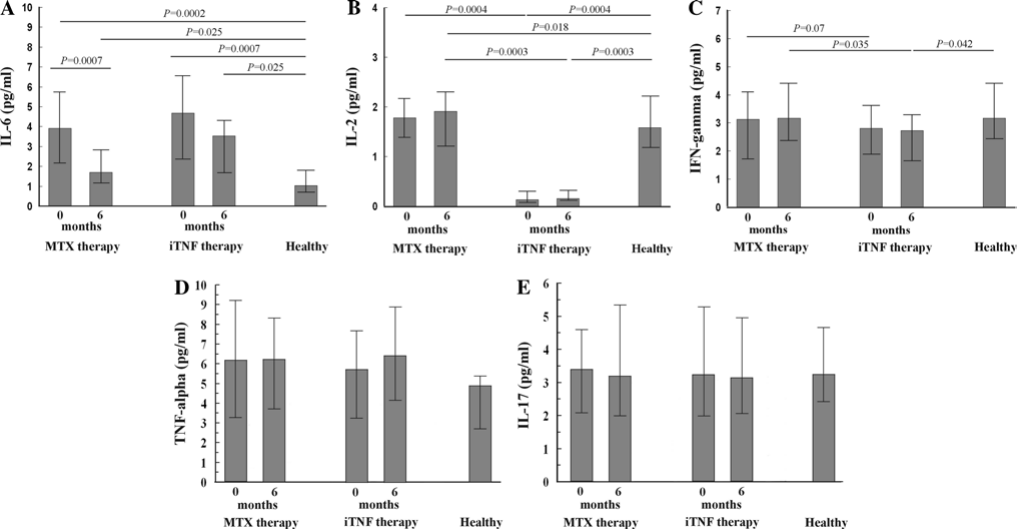
Serum cytokine concentrations in RA patients before and after the treatment compared to healthy controls. The median levels of cytokines. **a** IL-6. **b** IL-2. **c** IFN-γ. **d** TNF-α. **e** IL-17. *Error bars* are the IQ range

We also verified whether the different duration of active RA might modify the serum cytokine profile, thus affecting CD4 Th cell differentiation. All RA patients showed higher serum concentrations of IL-6 than controls, with the highest values in the iTNF group (Fig. 3a). Solely the iTNF group possessed lower levels of Th1 cytokines, more pronounced considering IL-2 (Fig. 3b, c). In contrast, no differences in Th1 cytokine values between MTX patients and controls were seen (Fig. 3b, c). Serum TNF-α and IL-17 reached similar levels in all patients and controls (Fig. 3d, e).

### Post-treatment distribution of PB Th1, Th17, and Treg cell population

Next, we tried to explore the relevance of therapy in the studied subpopulations depending on RA duration. A decrease in the PB Th17 cell population after the treatment in all patients, but more vigorous in MTX patients, was found (Fig. 2b). Nevertheless, the iTNF treatment up-regulated IL-17 gene activity, resulting in higher relative expression of IL-17 mRNA in CD4+ T cells of iTNF patients compared to the MTX group and to controls (Table 2). Accordingly, Th17 cells were expanded to twofold higher levels in PB of patients compared to controls; however, the differences were not statistically significant (Fig. 2b). Although the levels of relative mRNA expression of both IFN-γ and FoxP3 did not differ markedly between studied groups (Table 2), the iTNF patients maintained a systemic Th1 cell loss after the treatment (Fig. 2a). In contrast, MTX patients demonstrated a similar Th1 cell population compared to controls (Fig. 2a). Therapy changed, albeit nonsignificantly, the frequency of PB Treg cells in all RA patients, reversing, in consequence, defective Treg proportions in the iTNF group (Fig. 2c). Nevertheless, Tregs from these patients maintained CTLA-4 expression in the diminished proportions of cells compared to healthy corresponding Treg cells (Fig. 2d).

### Post-treatment serum cytokine profile

We assessed serum cytokine modification under the different therapeutic interventions regarding RA duration as well. A decline of serum IL-6 concentrations was seen only in the MTX group. Nevertheless, its level was not normalized in all patients (Fig. 3a). Among the patients, IL-6 concentration was highest in the iTNF group. IL-2 and IFN-γ remained at lower concentrations in sera from iTNF patients (Fig. 3b, c). In contrast, increased IL-2 levels were found only in the MTX group (Fig. 3b). Neither MTX nor iTNF treatment changed TNF-α or IL-17 concentrations in RA, their levels being comparable to those seen in controls (Fig. 3d, e).

### Correlation between clinical, immune, and laboratory parameters in patients at different stages of RA

We analyzed correlations among the proportion of examined T helper subpopulations (Th1, Th17, and Treg) in PB, serum soluble factors (IL-6, IL-2, IFN-γ, TNF, and IL-17), and clinical and/or laboratory features of RA in all studied groups of patients before and after the treatment. We found several statistically significant relationships among studied parameters, of which the most interesting comparisons have been presented in Table 3.

**Table 3:** Correlations between clinical, immune, and laboratory parameters in RA patients

Correlated parameters	MTX treatment		iTNF treatment	
	Before	After	Before	After
IL-6/CRP	*P* = 0.015 *r* = 0.61	*P* = 0.008 *r* = 0.62	*P* = 0.04 *r* = 0.53	*P* = 0.0004 *r* = 0.74
IL-6/Th1	NS	NS	*P* = 0.05 *r* = −0.45	NS
IL-6/Th17	NS	NS	*P* = 0.04 *r* = 0.66	NS
IL-6/DAS28	NS	NS	NS	*P* = 0.02 *r* = 0.54
Th1/DAS28	NS	NS	*P* = 0.03 *r* = −0.50	NS
Th1/CRP	NS	NS	*P* = 0.05 *r* = −0.45	*P* = 0.01 *r* = −0.56
Th1/Th17	NS	NS	*P* = 0.04 *r* = −0.69	*P* = 0.06 *r* = −0.47
Th1/Treg	NS	NS	NS	*P* = 0.05 *r* = 0.45
IL-2/CRP	NS	*P* = 0.007 *r* = −0.92	NS	NS
IL-2/CTLA-4+Treg	NS	NS	*P* = 0.02 *r* = 0.71	NS
Treg/CRP	NS	NS	*P* = 0.002 *r* = −0.87	NS
CTLA-4+Treg/CRP	NS	NS	NS	*P* = 0.02 *r* = −0.64
Th17/Treg	NS	NS	*P* = 0.05 *r* = −0.65	NS
CTLA-4+Treg/Th17	*P* = 0.01 *r* = − 0.61	*P* = 0.05 *r* = − 0.45	NS	*P* = 0.04 *r* = −0.44
TNF/Treg	NS	NS	*P* = 0.05 *r* = −0.65	NS
TNF/IL-17	*P* = 0.0000001 *r* = 0.95	*P* = 0.0000001 *r* = 0.95	*P* = 0.0002 *r* = 0.76	*P* = 0.01 *r* = 0.58
IL-2/IFN	NS	NS	NS	*P* = 0.05 *r* = 0.62

*NS* nonsignificant

## Discussion

Our present work strongly demonstrates that RA patients present an imbalance in CD4 T cell subsets’ distribution in PB, more pronounced in those with the most advanced disease, suggesting the impact of disease progression on subsequent changes. Considering CD4 T cell subpopulations, a common disorder found in all RA patients irrespective of the disease duration was expansion of Th17 cells in circulation. This observation is in accordance with previous reports [11–20] and confirms that Th17 is a key effector cell in the pathogenesis of RA [21]. Its strong pro-inflammatory action has been attributed to secretion of IL-17 and, to a lesser extent, TNF-α and IL-6 [22]. In addition, IL-17 induces the production of TNF-α, IL-6, and IL-1beta in numerous cell types [23] and can act in synergy with TNF-α to stimulate the production of pro-inflammatory cytokines, chemokines and metalloproteases from synovial fibroblasts, leading finally to cartilage loss [24, 25]. Moreover, during the course of RA it can promote osteoclastogenesis and bone absorption [26]. On the other hand, Th17 differentiation is dependent on IL-6 abundantly expressed in the inflamed joints and PB in patients with active RA, which emphasizes their reciprocal regulation [27, 28]. Permanently augmented concentrations of IL-6 in sera from RA patients [29] have been also confirmed in our study even after the biologic treatment. Consequently, we observed significantly enhanced activity of the IL-17 gene in the same patients. It should be stressed, however, that only patients with relatively short duration of active RA exhibited a higher potential for reversion of dysregulated IL-6 and Th17 cells as well as for reduction in disease activity compared to those with advanced disease.

Of note, no significant changes were observed in the serum levels of TNF-α and IL-17 in all patients at each time point tested, which was an unexpected observation when we consider their strong pro-inflammatory action and fundamental role in RA development [30]. Previous assessments demonstrated no significant increment [13, 14, 31] or, alternatively, an increase in TNF-α or IL-17 in sera from RA patients [11, 12, 30, 32–35]. It seems reasonable that unstable in vitro activity of TNF-α and IL-17 probably resulting from the relatively short half-life of both cytokines might affect their detectable levels found in our experiments [35]. Since IL-6 has a much longer half-time and is closely and positively interrelated with TNF-α and IL-17 in all studied patients, it appears to reflect intrinsic concentrations of both factors, thus being a better disease marker for measurements in patients’ sera [35].

It should be emphasized that progression of RA seems to correspond with many additional alterations in systemic distribution of T helper subsets studied, as was recently suggested [36]. In particular, we demonstrated that during disease evolution PB Th17 expansion was accompanied by diminished Th1 and Treg cell populations, indicating a profound imbalance in pro- and anti-inflammatory T cells in the periphery. We found that changes in the intracellular expression of IFN-γ, IL-17, and FoxP3 transcription factor seen in PB CD4+ T cells from untreated RA patients might be due to the post-transcriptional alterations rather than the markedly disturbed gene activity. Recently, other investigators have reported dysregulated Treg numbers in the periphery in active RA. Some studies demonstrated decreased [37], unchanged [38], or increased percentages of peripheral Tregs in the course of RA [39]. All these discrepancies may result from the differences in determination of Treg unique phenotype. Furthermore, since the CTLA-4 molecule plays a crucial role in Treg function, our study showing decreased CTLA-4+ Treg frequency in PB in advanced RA points to the possibility of Treg functional impairment especially during disease progression [38, 40]. However, the overall reasoning has to be cautious due to the lack of Treg functional assay in our experiment.

The current study clearly showing a systemic loss of Th1 cell population in progressive RA corresponds with some recent observations on a relation of RA activity/progression with Th1 defective response in PB [41, 42]. However, several previous reports showed contradictory results; some of them described increased [15, 35] or unchanged frequencies of Th1 cells in circulation in the course of RA [43, 44]. These discrepancies may be related to the fact that examined patients exhibiting a systemic decrement of Th1 cell frequency had much longer duration of the active disease, which may indicate the impact of RA progression on Th1 lineage restriction. In contrast, patients with relatively short-term disease assigned to MTX therapy exhibited no changes in the numbers of PB Th1 cells, which was consistent with past studies [43, 44].

Mechanisms underlying the decreased proportion of peripheral Th1 cells in advanced RA are not fully understood, but the influence of the serum cytokine profile affecting CD4 Th cell differentiation should have been taken into account. Since we found that the levels of cytokines exerting inhibitory activity toward Th1 differentiation, including IL-6 and IL-17 [45, 46], did not differ significantly between examined groups of patients, it seems that other factors may affect the Th1 lineage in progressive RA rather than dysregulated differentiation. In fact, a few previous reports suggested selective migration of Th1 cells from PB into inflamed joints in active RA [41, 47, 48], which might result in altered Th1 frequencies in PB. It cannot be excluded that the strength of this process corresponds with disease activity/severity, since we found that solely the iTNF group of patients remained with a systemic Th1 defect inversely correlated, in addition, with stably increased CRP levels even after the therapy. Another explanation for PB Th1 cell down-regulation in progressive RA is the possible impact of long-term use of some drugs capable of interfering with the Th1 phenotype, such as MTX, iTNF, and/or statins [49–51]. It is, however, difficult to distinguish between the role of pharmacological treatment per se and the severity/duration of RA in decrement of Th1 cell percentages as both parameters seem to be closely interrelated.

Consequently, the lower proportion of circulating Th1 cells was accompanied by diminished serum levels of Th1 cytokines, including IL-2 and IFN-γ. In fact, qualitative impairment of PB effector T cells, especially those with Th1 phenotype, was attributed to chronic exposure to stimulation via TNF-α [52]. Consistently with us, a deficit in serum Th1 cytokines in active RA and/or their negative relationship with disease activity have also been reported [34, 35, 41, 42, 53]. It is worthy of note, however, that our results contradict several previous reports showing increased or unchanged circulating Th1 cytokines in RA [12, 32, 33, 35] only in the part considering patients with the most advanced disease. Furthermore, in the same group of patients, we did not observe any influence of long-term iTNF treatment on changes in PB Th1 cell responses, as they entered the study with compromised baseline Th1 cytokine values. In some experiments, involving patients with relatively early stages of RA with no Th1 defects, a down-regulating effect of iTNF was observed [15, 50].

Given that IL-2 was demonstrated to be critical for the differentiation of Tregs from naïve CD4+ T cells, the lack of significant difference in Treg frequency between the MTX group and iTNF group despite evident decrease in serum IL-2 levels in the latter was, in fact, an unexpected finding. Nevertheless, it seems to be consistent with the recent studies showing an important role of IL-2 in the maintenance of homeostasis of the circulating Treg cell compartment [54], consisting of both the natural CD62L^hi^ and induced CD62L^lo^ FoxP3+Tregs [55]. It has been shown that natural Tregs are more sensitive to changes in the level of IL-2 than induced Treg cells [56]. In particular, Goudy et al. [56] demonstrated that IL-2 shortage could correlate with a decreased proliferative status of natural Tregs in a mice model of autoimmunity, but not with induced Tregs, despite similar levels of CD25 and CTLA-4 surface expression between the arms. Therefore, an obviously reduced expression of serum IL-2 found in iTNF patients with progressive disease might dysregulate the ratio of the CD62L^hi^ to CD62L^lo^ Tregs, which exert different suppressive potential, thus resulting in qualitative rather than quantitative differences in the FoxP3+ Treg pool detected in their circulation.

In addition to the generation and survival of Treg cells, inhibition of Th17 polarization appears to be an important function of IL-2; hence this Th1 cytokine is capable of controlling the Th17/Treg balance in the periphery [5, 57, 58]. In fact, an affected Th1 response has been suggested to shift the T cell homeostasis from a Treg-mediated tolerant state to Th17-mediated active inflammatory conditions [4, 57]. An increase in inflammatory disease activity in the presence of impaired Th1 responses has been well documented [6, 59–61]. Therefore, an increased Th17/Treg ratio in PB of the most advanced RA patients examined, which corresponded with either down-regulation of Th1 cells or laboratory signs of inflammation strengthens the suggestion that those systemic alterations may be, in fact, an immune signature of disease progression. In this context, we observed a Th1 protective response only in patients with short-term RA. Additional correlations among studied subpopulations and markers of inflammation seen in advanced RA emphasize a major role of systemic Th1 alterations in the management of Th17/Treg imbalance at this stage of the disease.

Herein, we also demonstrated that the main immune advantage of the therapy with MTX and/or iTNF in examined RA patients was the reversion of Th17 cell expansion. Available data concerning the effect of the different therapeutic interventions on the Th17 cell population are still confusing. Several studies have shown an unchanged [13, 15, 20] or, alternatively, a decreased Th17 frequency following treatment with MTX and/or iTNF [13–16, 19, 20], and the latter findings are in accordance with the current study. Similarly to the recent work [20], monotherapy with MTX was able to effectively limit the Th17 population only in the patients with early disease. In general, our study showed that the treatment used exhibited the most beneficial effect mainly for patients at a relatively early stage of the disease, as Th17 expansion was the only Th cell alteration seen in those patients’ circulation. As regards the patients with progressive disease, they required biologic treatment to be used. All the reports consistent with ours [13–16, 19, 20] strengthen the conclusion that the effectiveness of iTNF therapy concerning Th17 expansion should be evaluated not earlier than 12 weeks after biologics usage irrespective of the MTX administration.

Although the therapy with iTNF partially corrected the Th17/Treg imbalance seen in the most advanced RA, which was in line with other reports [15], it was accomplished also due to affecting the Th17 population, but not by evident Treg frequency improvement. Nonetheless, systemic Th1 loss and compromised Treg function, as evaluated by sustained lower PB Th1 and CTLA-4+ Treg frequency in the iTNF group despite the treatment, seemed to be irreversible phenomena in progressive disease. Although restoration of Treg suppressive activity after iTNF therapy has been previously reported, this improvement was only transient or restricted to the patients responding to the treatment [38]. We suggest that the compromised influence of iTNF on the proportion of functional CTLA-4+ Tregs in progressive RA is associated with an irreversible deficit in Th1 cytokine level. In particular, serum IL-2 shortage might be responsible for persistent qualitative alterations within Treg pool, when we consider a crucial role of IL-2 either in the maintenance of Treg compartment homeostasis [56] or in CTLA-4 expression in T cells [62]. While it is true that CTLA-4 contributes to the suppressive function of Tregs [40], it cannot be used as a single marker to define the functional status of a Treg; hence its functional significance should be considered with caution.

In conclusion, our study shows that both the degree of the immune alterations in PB of RA patients and their reversal after different therapeutic interventions depend on the duration of the active disease. Patients with relatively short-term disease possess a higher capacity for reversion of the studied abnormalities. In contrast, patients with progressive and long-term RA remain with much immune dysregulation managed by Th1 systemic defects even after iTNF treatment. Therefore, patients at early stages of active RA would certainly benefit from iTNF administration rather than those with the most advanced disease. In this context, new approaches based on restoration of Th1 protective responses capable of controlling the balance between Th17 and Tregs would be of interest as another target for the treatment of progressive RA. Recently, such a study concerning the influence of exogenous IL-2 on the balance in Th1, Th17, and Treg cell distribution in the different stages of RA was performed in our laboratory (data to be published).
